# Mental health system costs, resources and constraints in South Africa: a national survey

**DOI:** 10.1093/heapol/czz085

**Published:** 2019-09-23

**Authors:** Sumaiyah Docrat, Donela Besada, Susan Cleary, Emmanuelle Daviaud, Crick Lund

**Affiliations:** 1 Department of Psychiatry and Mental Health, Alan J. Flisher Centre for Public Mental Health, University of Cape Town, 46 Sawkins Road, Rondebosch, Cape Town, Western Cape, South Africa; 2 Health Systems Research Unit, South Africa Medical Research Council, Cape Town, South Africa; 3 School of Public Health and Family Medicine, Health Economics Unit, University of Cape Town, Cape Town, South Africa; 4 Health Service and Population Research Department, Centre for Global Mental Health, King’s Global Health Institute, Institute of Psychiatry, Psychology and Neuroscience, King’s College London, London, UK

**Keywords:** Health system costs, mental health system, health planning, mental healthcare, developing countries, policy implementation

## Abstract

The inclusion of mental health in the Sustainable Development Goals represents a global commitment to include mental health among the highest health and development priorities for investment. Low- and middle-income countries (LMICs), such as South Africa, contemplating mental health system scale-up embedded into wider universal health coverage-related health system transformations, require detailed and locally derived estimates on existing mental health system resources and constraints. The absence of these data has limited scale-up efforts to address the burden of mental disorders in most LMICs. We conducted a national survey to quantify public expenditure on mental health and evaluate the constraints of the South African mental health system. The study found that South Africa’s public mental health expenditure in the 2016/17 financial year was USD615.3 million, representing 5.0% of the total public health budget (provincial range: 2.1–7.7% of provincial health budgets) and USD13.3 per capita uninsured. Inpatient care represented 86% of mental healthcare expenditure, with nearly half of total mental health spending occurring at the psychiatric hospital-level. Almost one-quarter of mental health inpatients are readmitted to hospital within 3 months of a previous discharge, costing the public health system an estimated USD112 million. Crude estimates indicate that only 0.89% and 7.35% of the uninsured population requiring care received some form of public inpatient and outpatient mental healthcare, during the study period. Further, mental health human resource availability, infrastructure and medication supply are significant constraints to the realization of the country’s progressive mental health legislation. For the first time, this study offers a nationally representative reflection of the state of mental health spending and elucidates inefficiencies and constraints emanating from existing mental health investments in South Africa. With this information at hand, the government now has a baseline for which a rational process to planning for system reforms can be initiated.

**Key Messages**

- Low- and middle-income countries (LMICs) such as South Africa, contemplating mental health system scale-up embedded into wider health-sector transformations towards universal health coverage, require detailed, reliable and locally derived estimates on current resources and expenditures on mental health—the absence of which has limited their ability to initiate a sustained and rational approach to planning for the scale-up of mental healthcare. We have to develop and test a methodology to fill these gaps that can be applied to other LMIC settings.
- The study found that South Africa’s public mental health expenditure represented 5.0% of the total public health budget, with wide disparities between provinces. Inpatient care represented 86% of mental healthcare expenditure, with nearly half of total expenditure on mental health occurring at the psychiatric hospital-level. Almost one-quarter (24%) of mental health inpatients are readmitted to hospital within 3 months of a previous discharge, costing the public health system 18.2% of the total mental health expenditure.
- Crude estimates indicate that only 0.89% and 7.35% of the uninsured population of South Africa requiring care received some form of public inpatient and outpatient mental healthcare, respectively, during the study period. Further, mental health human resource availability, infrastructure and medication supply are significant constraints to the realization of the country’s progressive mental health legislation.
- For the first time, this study offers a nationally representative reflection of the state of mental health spending and elucidates inefficiencies and constraints emanating from existing mental health investments in South Africa. With this information at hand, the government now has a baseline for which a rational process to planning for system reforms can be initiated.

## Introduction

Over the past decade, calls to address the increasing burden of mental, neurological and substance use (MNS) disorders and to include mental healthcare as an essential component of universal health coverage (UHC) have attracted mounting interest from governments (Prince *et al.*, 2007; Mnookin, 2016; Chisholm *et al.*, 2019; Patel *et al.*, 2018). With the inclusion of mental health in the 2015 Sustainable Development Goals (SDGs), there is now a global commitment to include mental health among the highest priorities for investment as a health, humanitarian and development priority [Chisholm *et al.*, 2006; World Health Organization (WHO), 2013, 2016; Thornicroft and Patel, 2014].

International evidence has articulated the most promising, cost-effective options for reducing the contribution of mental disorders to the global burden of disease, particularly for low- and middle-income countries (LMICs; Patel *et al.*, 2018). Briefly, strategies include: the explicit recognition and inclusion of mental health in the UHC agenda; intensified investments in mental health systems; reducing inefficiencies in the use of resources through the redistribution of budgets from hospi-centric care to the community; task-shifting mental healthcare to non-specialist providers who receive ongoing specialist supervision; amplified training for all cadres of mental health professionals and specialists; the initiation of early interventions that are accessible to at-risk populations; integration of mental health in broader primary healthcare, and; the active engagement of those living with and effected by MNS disorders in the reform process (Chisholm *et al.*, 2007; Patel *et al.*, 2010; WHO, 2010; Lund *et al.*, 2012; Hanlon *et al.*, 2014; Thornicroft and Patel, 2014; Lund, 2016; Hanlon *et al.*, 2018; Patel *et al.*, 2018). With an expanding array of evidence-informed recommendations for scaling-up integrated mental healthcare, preventing mental illness and improving population mental health, coupled with an intensifying global momentum for investment; the question arises as to why there has been slow action in the way mental health services are financed and delivered (Chisholm, 2007).

Since the WHO Mental Health Atlas (MHA) initiative commenced in 2001, our understanding of mental health systems and ability to monitor progress towards the ambitious global mental health goals outlined in the SDGs has improved significantly (Chisholm *et al.*, 2006; WHO, 2013, 2016) Yet, significant gaps in the knowledge base remain among most LMICs, including South Africa. For example, among the 127 LMICs that were able to partially complete the WHO MHA (2017) questionnaire, only 40% (*n* = 51) were able to report on total government expenditure on mental health (WHO, 2018b). Service coverage estimates were reported by only 41% (*n* = 52) of LMICs (WHO, 2018b). The most common reason for missing data is that it simply does not exist, with a further limitation that most information provided by countries relates to the country as a whole, overlooking important variability across regions, concerning the degree of policy implementation, availability of services and the existence of promotion and prevention campaigns for mental health (WHO, 2018b).

LMICs such as South Africa contemplating mental health system scale-up, embedded into wider SDG- and UHC-related health-sector transformations, require detailed, reliable and locally derived estimates on current resources and expenditures on mental health—(1) as an indicator for measuring the efficiency of existing investments; (2) to measure inequities in resourcing and access; (3) to identify priorities and plan mental health services; (4) to provide a baseline against which additional resource needs estimates can be monitored; and (5) for targeting service reforms towards addressing the health system constraints that may limit scale-up efforts (Chisholm *et al.*, 2006; Saxena *et al.*, 2006; Chisholm *et al.*, 2017; Eaton and Ryan, 2017).

South Africa has taken some critical steps forward to strengthen its mental health system including reforming the Mental Health Care Act 17 of 2002 (MHCA), the development of the South African National Mental Health Policy Framework and Strategic Plan 2013–2020 (MHPF) and the adoption of the National Health Insurance (NHI) Policy (2017) to promote equity in health service delivery towards UHC [Department of Health (DOH), 2002, 2013, 2017]. South Africa’s health system currently comprises a large public sector that serves about 84% of the population and a smaller private sector which serves the affluent minority. Considering that only 40% of the overall health budget in South Africa is funded by the state—the public health system is under extreme pressure to expand healthcare access. In keeping with international human right standards, the MHCA introduces Mental Health Review Boards (MHRBs) and commits to the establishment of 72-h assessment areas in district-level general hospitals before referral to specialist mental hospitals (Lund *et al.*, 2011).

Despite the country’s comprehensive MHPF and MHCA, health budgets and broader health sector transformations have not followed to actualize the contents of the policy (DOH, 2013; Jack *et al.*, 2014). Progress in service delivery is challenged by inadequate usage of national-provincial dissemination channels to communicate and promote the MHPF and MHCA, a lack of technical support around policy implementation within provinces, as well as a weak health information system leading to a lack of information about the true burden of MNS disorders, patterns of mental health service access, human resources (HR) for mental health, and provincial and national budgets for mental health services rendered outside of the specialized (psychiatric) care levels (Lund *et al.*, 2010; Lund *et al.*, 2011; Schneider *et al.*, 2016; Chisholm *et al.*, 2017; Docrat *et al.*, 2019). Further, with no explicit reporting requirements linked to the MHPF and MHCA, the degree to which they have been implemented remains unknown. Without explicit understanding of these aspects of the current mental health system and its resource environment, the active integration of mental health into the future health system of South Africa along with and the achievement of the MHPF will be challenging (Docrat *et al.*, 2019). For this reason, South Africa represents an ideal LMIC setting to develop and test a methodology to fill this gap that can be applied to other settings.

The aim of this article is to quantify public health system expenditure on mental health services, by service-level and province, and to document and evaluate the resources and constraints of the mental health system in South Africa in order to inform a rational approach to planning effectively for mental health service scale-up.

## Materials and methods

### Setting

This study was conducted across all nine provinces of South Africa at all levels of the public health system and reports the full costs of mental health services and programmes rendered through the Provincial and National Department(s) of Health (NDOH) between 1 April 2016 and 31 March 2017 [i.e. the 2016/17 financial year (FY)]. The population in need is assumed to be equivalent to those without private health insurance, who typically depend on the public health system for their care.

### Costing approach, perspective and time frame

This study employed a cross-sectional, accounting-based, aggregate costing approach using primary and secondary data sources (Barnum and Kutzin, 1993; Stenberg and Rajan, 2016). This method is appropriate given that the aim was to assess the total cost of mental health services rendered by all health facilities, at all levels of the public health system in South Africa and more detailed costing approaches would not have been feasible in light of data availability and the lack out routine information systems for mental health in the country (Barnum and Kutzin, 1993; Razzouk, 2017). The cost analyses were conducted from the provider perspective. All costs are expressed in 2016/17 US Dollars (USD).

### Data collection and data sources

#### Primary data collection and study sample

Data collection took place between January and October 2018. Three data collection tools were designed purposively for three categories of respondents: (1) Provincial Departments of Health (PDOH); (2) Regional, Tertiary, Central and Specialized Hospitals, and; (3) Primary Health Care (PHC) facilities and District Hospitals (District health system). Each instrument was sent directly to the target respondents via PDOH. The e-mailed instruments were followed up with telephone calls and ongoing support to all respondents. Table 1 outlines the key domains of each instrument, a description of the respondents, the sample size for each province and the overall response rate(s) achieved.

**Table 1.** Overview of primary data collection tools, respondents and sample sizes, by province

	Provincial department of health data collection	Regional, tertiary, central and specialized hospital data collection					PHC and district hospital data collection
Key domains of data collection instrument	Provincial-level financial allocations to different service levels Subsidies for contracted mental health services Subsidies and service descriptions regarding day and residential care for MHCU	Mental health HRs Medication availability and stock-outs Outpatient and inpatient mental health visits Average length of inpatient mental health admissions Patient load by mental health disorders Readmission rates for MHIAs					Mental health HRs Medication availability and stock-outs Characteristics of designated district hospital 72-hour assessment areas Outpatient and inpatient mental health visits Mental health prevention and promotion campaigns Residential and day care facilities.
Respondents	Provincial director(s) of non-communicable disease, director(s) of mental health and/or mental health co-ordinators	Hospital directors and chief executive officers, psychiatrists, pharmacists, operational managers and nursing managers					District health service co-ordinators and district mental health co-ordinators
Organizational level	Provincial offices	RHs	THs	CHs	SPHs	OSHs	Health districts (PHC facilities and DH)
National target sample size	9	47	18	9	24	6	52
Sample sizes, by province							
Eastern Cape	1	2			3		7
Free State	1	2			1		5
Gauteng	0	5	2	1	3	1	5
KwaZulu-Natal	1	6	2	1	5		10
Limpopo	1	3	2		3		5
Mpumalanga	1	3	2				3
North West	1	1	1		1		0
Northern Cape	1	2			2		5
Western Cape	1	1	1	2	4		2
National sample size	8	25	10	4	22	1	42
Response rate	88.9%	53.2%	55.6%	44.4%	91.7%	16.7%	80.8%

PHC facilities, Health Posts, Mobile Clinics, Clinics, CDCs, CHCs; DH, District Hospital; RH, Regional Hospital; TH, Tertiary Hospital; CH, Central Hospital; SPH, Specialized Psychiatric Hospital; OSH, Other Specialized Hospital.

At the provincial-level, completed provincial data collection tools were received from 8/9 PDOH in South Africa, with 1 PDOH submitting a partially completed provincial data collection tool (Table 1). For hospitals, response rates were 53.2%, 55.6%, 44.4%, 91.7% and 16.7% for regional, tertiary, central, specialized psychiatric and other specialized hospitals, respectively. This represented 62 of 104 hospitals in the country. At the district level, 42 data collection tools were received from the 52 health districts of South Africa, representing a response rate of 80.8%. The sample size generated through primary data collection was supplemented with a number of secondary datasets (outlined below) to allow for costs to be appropriately modelled for all facilities and health districts in the country. Although total health system mental health expenditure was estimated for all public sector facilities in the country, the evaluation of mental health system resources and constraints (e.g. medication availability, readmission rates, duration of inpatient mental health admissions and district hospital infrastructure for mental health) was limited to the sample of facilities that completed primary data collection.

#### Secondary sources

Several secondary data sources were used in this study (Supplementary Table S1). The District Health Information System (DHIS) data file supplied by the NDOH provided age-disaggregated indicators of total mental health outpatient visits (MHOVs) and mental health admissions by the facility. The Health Systems Trust District Health Barometer (HST–DHB; 12th Edition—2016/17) data file (Massyn *et al.*, 2017) provided: hospital-level indicators of expenditure per patient day equivalent (PDE) for all categories of hospitals; and indicators of expenditure per PHC headcount for all health districts for the 2015/16 FY. Costs from the 2015/16 FY were converted to real 2016/17 prices using the Consumer Price Index of 6.8% obtained from Statistics South Africa (STATS SA 2017). Data quality of the DHIS is addressed through checking of the data for inaccuracies by clinic managers and supervisors, using minimum and maximum expected values for data elements, and using the DHIS software. However, it is known that in many health facilities there are a number of barriers to efficient and accurate reporting that cast doubt on the reliability and validity of these data. The DHB produced by the HST provides a detailed overview of the country’s public health services in all 52 health districts. The publication has become an important planning and management resource for health service providers, managers, researchers and policy-makers in the country. The compilation of the DHB is guided by a technical work group made up of managers from the NDOH and HST. The NDOH Average Length of Stay (ALOS) data file, supplied by the Parliamentary Monitoring Group, provided the Average Length of (inpatient) Stay for each hospital in South Africa, organized by province (Average Length of Stay, 2018). The NDOH Personnel and Salary System (PERSAL) database was obtained to estimate mental health staffing coverage.

### Data management and analysis approach

A linked Excel database was created for storing all data. The calculations performed to arrive at the cost estimates are described below. The results are presented by each category of facility, and by inpatient and outpatient costs. Age-disaggregated costs are provided for outpatient visits for adults (18 years and older) and children (under 18 years).

#### Hospital-level cost analysis

In order to estimate mental healthcare inpatient and outpatient costs at the hospital level, the inpatient and outpatient estimates of cost per PDE were multiplied against inpatient and outpatient mental health utilization data across all hospitals (Box 1). Where the total number of mental health inpatient admissions (MHIAs) and/or MHOVs is not provided directly from the facility, these data were included from the DHIS. Where facilities directly provided these data, and the totals as reported by the DHIS were either higher or lower, we systematically used the higher estimate to ensure that costs were not underestimated. The variation between each data source was not substantial.

**Box 1. Methods and Data Sources for the Calculation of Inpatient and Outpatient Mental Health Costs**

**Inpatient Costing**

$$\text{Total}\;\text{Inpatient}\;\text{Cost}={\text{Expenditure}\;\text{per}\;\text{Patient}\;\text{Day}\;\text{Equivalent}}^{a}\;{\times \;\text{Total}\;\text{Inpatient}\;\text{Days}}^{b}$$

$$\text{Total}\;\text{Inpatient}\;\text{Days}={\text{Inpatient}\;\text{Admissions}}^{c}\;{\times \;\text{Average}\;\text{Length}\;\text{of}\;\text{Stay}}^{d}$$

^a^Cost per Patient Day Equivalent (PDE) was drawn from the DHB 2016/17 data file for each facility (Massyn et al., 2017). These estimates were provided up until the financial year ending 2015/16. We adjusted the the 2015/16 estimates to real 2016/17 prices using the Consumer Price Index of 6.8% (STATS SA, 2017).

^b^Total Inpatient Days was calculated by multiplying Total MHIA between 1 April 2016 and 31 March 2017 by the ALOS for these inpatients between 1 April 2016 and 31 March 2017. It was assumed that the inpatient days of existing patients at the beginning of the year will balance out the inpatient days of patients admitted towards the end of the year who would be discharged in the following year.

^c^Inpatient admission data was drawn from primary data provided by facilities or from the DHIS using the indicator *Mental health admissions total*. If the DHIS and primary data collection responses differed, we used the higher reported figure.

^d^ALOS data was drawn from the primary data collection responses from each hospital. Hospitals reported the ALOS (in days) across all MHIA between 1 April 2016 and 31 March 2017. For facilities that were not able to specify an ALOS for MHIAs, the ALOS for all admissions was used, and a sensitivity analysis was performed based on the average difference between LOS for all admissions and mental health admissions, by level of service. When average length of stay exceeded one year, a maximum length of stay of 365 days was applied.

**Outpatient (OPD) Costing: Hospital-Level**

$$\text{Total}\;\text{Outpatient}\;\text{Cost}={\frac{\text{Expenditure}\;\text{per}\;\text{Patient}\;\text{Day}\;\text{Equivalent}}{3}}^{e}\times Total OPD Visits by Mental health Clients$$

**Outpatient (OPD) Costing: PHC-Level **

$$\text{Total}\;\text{Outpatient}\;\text{Cost}={\text{Expenditure}\;\text{per}\;\text{PHC}\;\text{Headcount}}^{f}\;\times \;\text{Total}\;\text{OPD}\;\text{Visits}\;\text{by}\;\text{Mental}\;\text{Health}\;\text{Clients}$$

^e^Total Mental Health Clients was drawn from the DHIS for each Mobile, Primary Care Clinic, Community Health & Day Center facility using the indicator *Mental health clients total*. Entries for the period April 1 2016 to 31 March 2017 were summed for each facility.

^f^Expenditure per Headcount was drawn from the District Health Barometer 2016/17 Data file for each Primary Care Clinic, Community Health & Day Center (Massyn *et al.*, 2017). We adjusted the 2015/16 estimates to real 2016/17 prices using the Consumer Price Index of 6.8% (STATS SA, 2017).

Inpatient days were calculated by multiplying the number of MHIAs within the reporting period by the ALOS for MHIAs, provided directly from facilities. Total inpatient expenditure was then calculated by multiplying inpatient days by the cost per inpatient PDE for each facility. When ALOS exceeded 1 year, a maximum length of stay of 365 days was applied as this study sought to estimate mental health expenditure over a 1-year period. It was assumed that the inpatient days of existing patients at the beginning of the year would balance out the inpatient days of patients admitted towards the end of the year that would be discharged in the following year.

As a number of hospitals across the country did not complete the primary data collection tools, we did not have mental-health-specific ALOS for every hospital in South Africa. For these hospitals, we first extracted the ALOS for all admissions from the NDOH ALOS database, which provided hospital-specific ALOS for 2017, and multiplied the number of MHIA within the reporting period by these ALOS estimates (Average Length of Stay, 2018). Using primary data from participating hospitals at each service-level, we then determined the average difference between ALOS for all inpatient admissions and ALOS for mental-health-specific admissions, for each service-level. A sensitivity analysis was then performed among hospitals for which mental-health-specific ALOS were not available, by adjusting the ALOS for all admissions based on the average difference between the duration of mental health vs all admissions for each level of care. The final inpatient cost for these hospitals (i.e. those with imputed ALOS) was reported as the mid-point between the total cost with and without sensitivity adjustment. No sensitivity analyses were performed for hospitals that provided a mental-health-specific ALOS.

Consistent with other empirical cost studied using the PDE methodology, outpatient expenditure at the hospital-level was calculated by multiplying the number of MHOV within the reporting period, as reported in the DHIS or through primary data at the facility-level, by one-third the cost per PDE for inpatients. This calculation assumes that the resources required to treat one outpatient represent one-third of the resources for treating a single inpatient (Massyn *et al.*, 2017).

For the assessment of the cost of readmissions, each hospital was asked to indicate the number of inpatient mental health patients that were readmitted as mental health inpatients within 3 months of a previous discharge. Costs of readmissions were then determined on a proportional basis, i.e. the proportion of inpatient admissions that were readmissions were applied to the total cost of inpatient admissions for each hospital to determine the total cost of readmissions. Where hospitals did not provide the total number of readmissions, we applied an average readmission rate for each hospital level in each province based on those that had completed primary data collection.

#### Primary healthcare-level outpatient cost analysis

Outpatient mental health expenditure at the PHC-level, which included mobile clinics, PHC clinics, Community Health Centres (CHCs), and Community Day Centres (CDCs), was calculated by multiplying the expenditure per PHC headcount for each health district obtained from the HST–DHB (Massyn *et al.*, 2017), by the total number of MHOV within the reporting period. More information about the differences in the types of clinics and the populations they serve can be found in Supplementary Table S2.

#### Non-governmental organization and contracted hospital cost analysis

Although all PDOH were asked to outline detailed information regarding financial transfers made for contracted hospital and non-governmental organization (NGO) mental health services within their provinces, including the name of facility, type of services rendered, number of inpatient and day patients, and the cost per patient day; none were able to comprehensively specify and validate the range of services and total financial transfers for these services. In lieu, we then requested PDOH to provide the overall total amount transferred for contracted hospital and NGO mental health services during the 2016/17 FY. For those that were able to provide this information, the absolute amount was used and total mental health expenditure was, therefore, expressed both including and excluding contracted hospital and NGO services for both national and provincial-levels

#### Financial adjustments

All costs were calculated in 2016/17 South African rands (ZAR) and were converted to 2016/17 USD based on the historical rates of exchange for the 2016–17 FY, reported by the United States Treasury (USD1 = ZAR13.6; United States Bureau of the Fiscal Service, 2018).

#### Analysis of mental health HRs, medication availability and infrastructure

For the assessment of public sector mental health HR availability, we relied on the NDOH PERSAL database of staffing as in August 2018, for all cadres except for psychiatrists. The number of public sector psychiatrists was obtained from primary data collection, and due to incomplete facility inputs, may reflect an underestimate in the number of these posts. The total number of mental health HRs were divided by the uninsured population in each province for the 2016/17 FY, and expressed as rates per 100 000 uninsured population. Given that the staffing data were for 2018, the estimates of uninsured populations for each province, obtained from the HST–DHB, were increased by a factor of 2% to account for population growth.

For the assessment of mental health medication stock-outs and infrastructure, we relied entirely on direct facility reports. All medications outlined for the treatment of MNS disorders were extracted from the Standard Treatment Guidelines (STG) and Essential Medicines Lists (EML) for each service level (DOH, 2012a,b). Hospitals and PHC facilities were requested to indicate whether, in the past 1 year, any of the listed medications for their service level were stocked-out or whether the medication was considered to be not-routinely available (NRA). Where stock-outs were reported, hospital(s) and PHC facilities indicated the duration of each stock-out. Due to the significant number of medications included in the instrument, the analysis of these data focused on summarizing the most frequently reported medications stocked at each level of care.

For the assessment of infrastructure, in line with the priorities outlined by collaborators at the NDOH, we focused our analysis on the degree to which designated district hospitals across the country have met the infrastructural criteria outlined by the MHCA (2002) and accompanying guidelines for the admission of mental health patients without consent for 72-h observation (DOH, 2002, 2012). Whilst the guidelines include a vast number of infrastructural requirements including close circuit television monitoring and panic buttons for staff, we prioritized the following criteria: whether district hospitals had a designated inpatient psychiatric unit; whether mental health inpatients are kept together with non-mental health patients in a general ward; whether adolescent and adult mental health inpatients are kept together, and; whether male and female mental health inpatients are kept separate from one another. These criteria are considered the most paramount for ensuring that the rights and dignity of users that cannot give consent and are posing a danger to themselves and others are protected. For each health district, contributors were asked to indicate which of their listed district hospitals were designated by the MHCA (2002) to admit mental health users for involuntary admission. Amongst these, contributors were then asked to indicate which of the listed criteria had been met. Responses were then summarized by the province.

### Ethical approval

This study made use of secondary data and collected routine health services data pertaining to mental health service delivery in South Africa from the NDOH and nine PDOH. No direct access to any facilities was required and no data that were collected in this study contained any patient identifiers. Ethics approval was obtained from the University of Cape Town Human Research Ethics Committee (HREC 744-2017) and from Provincial Health Research Committees (PHRC) in each province. Written permission for this study was also provided by Provincial Heads of Health.

## Results

### Health system costs of mental health services

This study found that the total health system costs of inpatient and outpatient mental health services across all provinces of South Africa amounted to an estimated USD573.6 million in the 2016/17 FY (Table 2). At the national level, this represented 4.6% of the total health budget (South Africa National Treasury, 2018) and equated to USD12.4 mental health expenditure for inpatient and outpatient care, per capita uninsured (i.e. for those without private health insurance who are assumed to be dependent on the public health system). When including transfers for contracted hospital and NGO mental health services, the total health system cost of mental health services increased to USD615.3 million or USD13.3 per capita uninsured. It must be noted, however, that not all provinces were able to comprehensively specify and validate the range of services and total financial transfers made for contracted hospital and NGO mental health services, and we have, therefore, expressed the results both including and excluding contracted hospital and NGO services for both national and provincial levels.

**Table 2:** Provincial and national summary of total costs of mental health services

	EC	FS	GT	KZN	LP	MPU	NC	NW	WC	National
Inpatient cost of mental health services (USD, millions)	50.8	16.4	152.9	110.8	21.8	10.0	10.7	18.8	100.0	492.1
Outpatient cost of mental health services (USD, millions)	8.5	2.2	18.8	23.8	9.3	3.1	2.3	3.0	10.5	81.5
Total inpatient and outpatient mental health service cost (USD, millions)	59.3	18.6	171.6	134.7	31.1	13.1	13.0	21.8	110.6	573.6
Total inpatient and outpatient mental health expenditure per capita (Uninsured; USD)	9.7	7.8	17.1	14.1	5.9	3.5	12.9	6.7	22.1	12.4
Proportion of 2016/17 health budget spent on mental health inpatient and outpatient services (%)	4.0%	2.8%	6.2%	5.0%	2.6%	1.7%	3.9%	3.1%	7.5%	4.6%
Total transfers for contracted hospital services for mental health (USD, millions)	8.9	*0.0* ^a**^	*0.0* ^a**^	11.3	*0.0* ^a**^	3.1	*0.0* ^a**^	*0.0* ^a**^	*0.0* ^a**^	23.3
Total DOH transfers to mental health NGOs (USD, millions)	0.8	0.2	13.7	1.0	*0.0* ^a**^	*0.0* ^a**^	*0.0* ^a**^	*0.0* ^a**^	2.7	18.4
Total costs of inpatient and outpatient mental health services and transfers to contracted hospitals and NGOs for mental health services (USD, millions)	69.0	18.7	185.3	147.0	31.1	16.1	13.0	21.8	113.3	615.3
Total costs of inpatient and outpatient mental health services and transfers to contracted hospitals and NGOs for mental health services per capita uninsured (USD)	11.3	7.9	18.5	15.4	5.9	4.3	12.9	6.7	22.6	13.3
Proportion of 2016/17 health budget spent on mental health inpatient and outpatient services and transfers to contracted hospitals and NGOs (%)	4.6%	2.8%	6.7%	5.5%	2.6%	2.1%	3.9%	3.1%	7.7%	5.0%

At the time this report was prepared, no PDOH were able to validate that the reported total transfers to contracted hospitals and NGOs represented *all* transfers to contracted hospitals and NGOs for mental health services in their respective provinces for the 2016/17 FY.

a Province was not able to comprehensively specify the total transfers for DOH contracted hospital and/or NGO services for mental health.

EC, Eastern Cape; FS, Free State; GT, Gauteng; KZN, Kwa-Zulu Natal; LP, Limpopo; MP, Mpumalanga; NC, Northern Cape; NW, North West; WC, Western Cape.

Per capita expenditure (uninsured) on inpatient and outpatient mental health services (i.e. excluding contracted hospital and NGO mental health services) ranged from USD3.5 in Mpumalanga to USD22.1 in the Western Cape. The North West, Limpopo, Free State and Eastern Cape provinces spent less than USD10.0 per capita (uninsured) on mental health inpatient and outpatient care. After the Western Cape, Gauteng and KwaZulu-Natal spent the most on inpatient and outpatient mental healthcare, with estimates of USD17.1 and USD14.1 per capita (uninsured), respectively; these provinces were the only three provinces (of nine) to spend 5.0% or more of their provincial health budgets on inpatient and outpatient mental health services. This trend was consistent when including expenditure on contracted hospital and NGO mental health services.

At the national level, 86% of overall health system expenditure on mental health was attributed to inpatient care, whereas the remaining 14% was attributed to outpatient care (Supplementary Figure S1; Table 2). This trend was consistent across all provinces in the country. Limpopo and Mpumalanga spent the highest share of their mental health expenditure on outpatient care: 29.8% and 23.8%, respectively. The lowest proportion of overall spending on outpatient care was seen in the Western Cape, where only 9.5% of the total mental health spending of inpatient and outpatient mental health services was spent on outpatient care.

**Figure 1:**
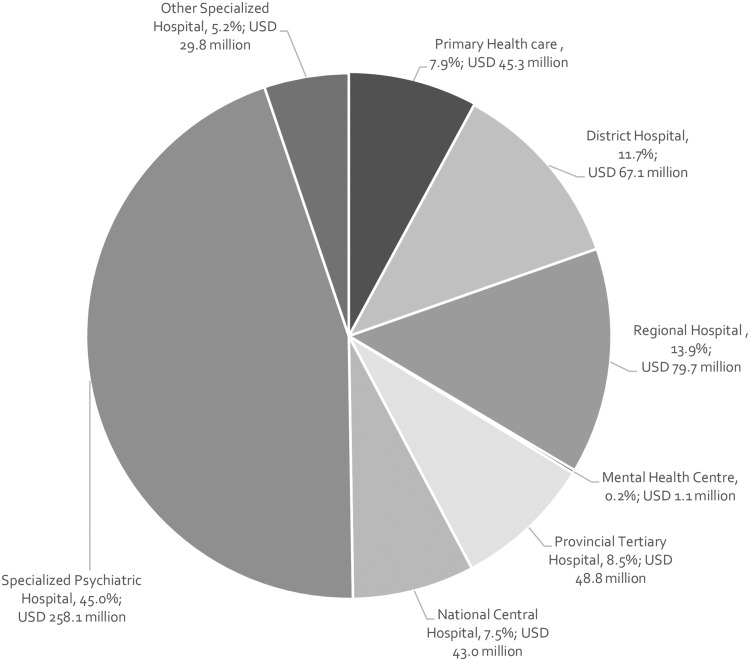
Distribution of total costs of inpatient and outpatient mental health services by service-level.

National-level estimates show that care at the specialized psychiatric hospital-level made up the large majority of the total cost (Figure 1), amounting to 45% of the total; with PHC-level mental healthcare accounting for 7.9%, district hospital mental healthcare accounting for 11.7% and, regional, tertiary and central hospital mental health services accounting for 13.9%, 8.5% and 7.5% of the total cost of inpatient and outpatient mental healthcare, respectively.

### Mental health readmission rates and costs

**Figure 2:**
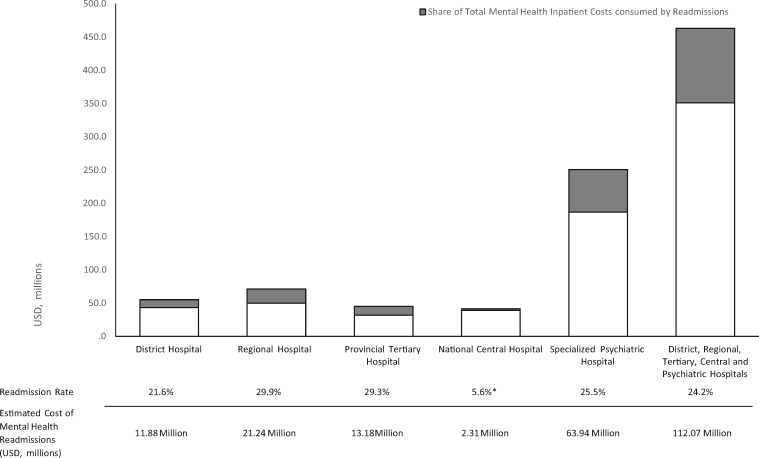
Readmission rates and estimated costs of mental health readmissions for district, regional, tertiary, central and psychiatric hospitals. *Only one National Central Hospital was able to provide the total number of mental health inpatients that were readmitted within 3 months of previous discharge.

Based on national average readmission rates obtained directly from facilities, across all hospital levels, the average overall readmission rate within 3 months from previous discharge for MHIAs was 24.2% (Figure 2). The service-level readmission rates for MHIA at district, regional, tertiary, central and specialized psychiatric hospitals were: 21.6%, 29.9%, 29.3%, 5.6% and 25.5%, respectively. Based on the inpatient cost calculations for each service level, readmissions during the 2016/17 FY are estimated to have cost approximately USD11.9 million at the district hospital level, USD21.24 million at the regional hospital level, USD13.2 million at the tertiary hospital level, USD2.3 million at the central hospital level and USD63.9 million at the specialized psychiatric hospital level. Using an average readmission rate for all service-levels, in total, readmissions cost the South African health system USD112.6 million, or 18.2% of the total mental health expenditure.

### Duration of MHIAs

**Figure 3:**
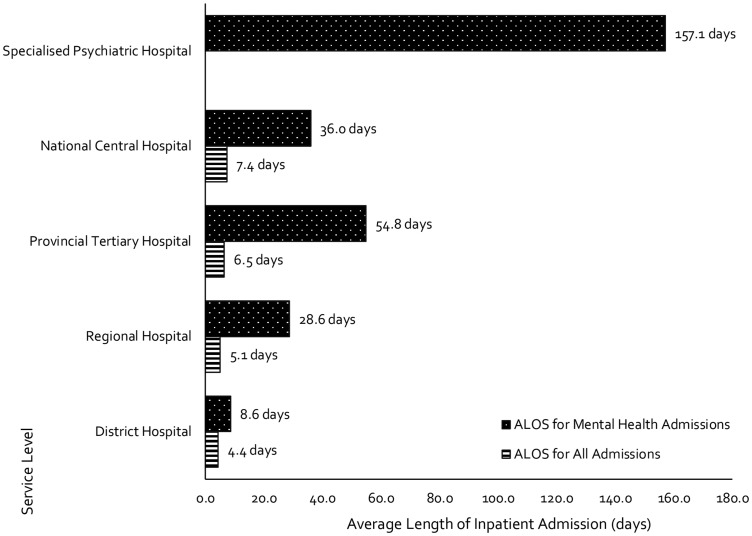
Average length of inpatient stay (ALOS) for all admissions vs mental health admissions, by service-level.

Across all hospital levels, MHIAs were found to be substantially longer when compared with the ALOS for all admissions (Figure 3). At the district hospital level, mental healthcare users (MHCUs) admitted for inpatient care spent twice as long in the hospital in comparison to all patients. At the regional and tertiary hospital level(s), MHIAs lasted nearly 6 and 8 times longer, respectively, when compared with inpatient admissions for all health conditions. At the central hospital-level, mental health patients admitted for inpatient care spent almost five times longer in hospital. Although all patients admitted at the specialized psychiatric hospital level were considered MHIAs, the ALOS at this level of care was 157.1 days.

### Mental health HRs

At the national level, this study found that there is an average 0.31 public sector psychiatrists per 100 000 uninsured population; with the Western Cape reporting the highest availability of psychiatrists at 0.89 per 100 000 uninsured population and Mpumalanga reporting the lowest rate, at 0.08 psychiatrists per 100 000 uninsured (Table 3). There remains a critical shortage of child psychiatrists with only three of the nine provinces of South Africa, namely the Western Cape, Free State and Gauteng, reporting any child psychiatrists working in the public sector.

**Table 3:** Mental health HRs per 100 000 uninsured population, by province

	EC	FS	GT	KZN	LP	MP	NC	NW	WC	National
Psychiatrist^a^	0.10	0.59	0.51	0.12	0.15	0.08	0.40	0.12	0.89	0.31
Sessional psychiatrist^a^	0.02	0.00	0.00	0.06	0.00	0.00	0.00	0.03	0.00	0.02
Psychiatry registrar^a^	0.00	0.00	0.00	0.00	0.00	0.00	0.00	0.00	0.12	0.01
Child psychiatrist^a^	0.00	0.04	0.02	0.00	0.00	0.00	0.00	0.00	0.08	0.02
Child psychiatry registrar^a^	0.00	0.00	0.00	0.00	0.00	0.00	0.00	0.00	0.08	0.01
Psychologists^b^	0.87	0	1.38	0.61	1.22	0.7	3.28	0.46	1.22	0.97
Psychologist (community service)	0.2	0.42	0.58	0.17	0.09	0	0.5	0	0.3	0.26
Psychologist intern	0.02	0.17	0.39	0.09	0.11	0.05	0.6	0	0.16	0.16
Medical officers	18.91	15.73	17.97	20.98	16.01	14.8	24.76	15.35	19.93	18.3
Medical officer (community service)	2.07	2.73	2.38	2.16	2.82	4.08	7.06	5.15	4.07	2.98
Medical officer (intern)	5.44	7.32	8.99	7.79	3.99	3.71	6.36	6.77	6.52	6.71
Occupational therapist (grades 1–3)	1.38	0	1.62	0.79	2.5	1.45	3.68	0.98	2.61	1.53
Occupational therapist (community service)	0.57	0.76	0.86	0.53	0.24	0.67	1.59	0.67	0.3	0.61
Speech therapists and audiologists (grades 1–3)	0.67	0	1.69	0.75	1.35	1.61	2.09	0.64	0.76	1.07
Social worker	1.9	0	2.44	2.07	0.64	1.26	2.98	1.41	2.65	1.83
Professional nurse	117.9	0	74.82	81.74	97.97	87.8	78.45	78.56	55.23	80
Professional nurse specialty	26.27	0	27.58	37.49	31.82	22.57	16.9	17.71	27.89	27.23
Professional nurse (community service)	10.21	9	7.19	7.31	1.66	5.91	10.64	13.36	7.16	7.47

a No data were available through the NDOH PERSAL database regarding total number of psychiatrists working in the public sector. These estimates are, therefore, based on responses received through primary data collection only and may be underestimated.

b The PERSAL database does not differentiate between Clinical Psychologists and other Psychologists. These figures, therefore, include the total number of psychologists (grades 1–3), senior clinical psychologists and principal psychologists (grade 1–3). It is assumed that a Masters’ degree in clinical psychology and registration with the health professions council of South Africa is a requirement for these posts.

EC, Eastern Cape; FS, Free State; GT, Gauteng; KZN, Kwa-Zulu Natal; LP, Limpopo; MP, Mpumalanga; NC, Northern Cape; NW, North West; WC, Western Cape.

There were 0.97 public sector psychologists, senior clinical psychologists and principal psychologists per 100 000 uninsured population. The availability of auxiliary health workers, critical for rehabilitative care and support services for MHCUs, was also found to be scarce with estimates of 1.53 public sector occupational therapists; 1.07 public sector speech therapists and audiologists, and 1.83 social workers per 100 000 uninsured population. The study also reported good coverage of nurses with 80 per 100 000 professional and 27.2 specialist nurses. These, however, may not all be psychiatric nurses.

### Mental healthcare utilization among adults, adolescents and children

**Figure 4:**
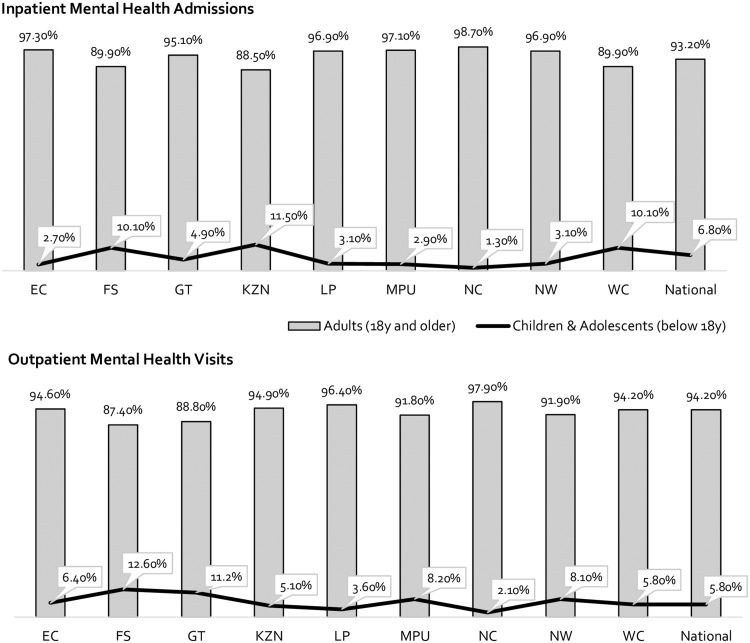
MHIAs and outpatient visits for adults, adolescents and children, by province, April 2016 to March 2017.

Collectively, 93.2% of MHIAs in South Africa were for adults aged 18 and older, with only 6.8% of MHIAs being recorded for those below 18 years (Figure 4). This trend was consistent across all provinces, with the highest rates of MHIAs for children and adolescents recorded in KwaZulu-Natal at 11.5%. Similarly, the proportion of adults aged 18 years and older receiving outpatient mental healthcare in the country represented 94.2% of all MHOV, compared with only 5.8% for those under 18 years. In the Free State, MHOVs for children and adolescents aged below 18 years accounted for 12.6% of all MHOVs, compared with only 2.1% in the Northern Cape.

### District hospital infrastructure for mental health

Despite the majority of district hospitals being designated by the MHCA (2002) for the provision of 72-h assessments; this study found that there are specific characteristics outlined by the MHCA (2002) that are not met across a large number of these hospitals (DOH, 2002). Although the North West and Western Cape provinces did not submit complete data regarding district hospital infrastructure, among the remaining provinces, over 62% of district hospitals indicated that adult mental health inpatients are kept in general wards with other patients, contrary to guidelines within the MHCA (Supplementary Table S3). The exception to this is in the Free State, which indicated that all its hospitals keep their mental health patients separately. KwaZulu-Natal, Limpopo, Mpumalanga and the Northern Cape indicated that over 80% of their district hospitals keep their mental health patients together with other patients. Furthermore, an extremely low proportion of district hospitals keep their adult and adolescent patients separately (13%), however, close to 80% of all district hospitals sampled separate female and male mental health inpatients.

### Mental health medication stock-outs

With respect to mental health medication availability, the findings illustrated that the most frequently stocked out medications are those prescribed for the treatment of adult depression and dysthymia, bi-polar disorder, psychosis, epilepsy, dementia, child and adolescent developmental disorders and adolescent behavioural-conduct disorder (Supplementary Figure S2). Starkly, among the sampled specialized psychiatric and regional hospitals, lithium was among the MNS medications most frequently reported as stocked-out. Further, at the district and regional hospital level(s), fluoxetine, the first-line treatment for major depressive disorders as per the STGs, was among the most frequently stocked-out. Both these drugs are listed as essential medicines by the WHO as well as several others listed in Supplementary Figure S2.

### MNS disorder prevalence and modelled (crude) estimates of access to care

The Global Burden of Disease [Global Burden of Disease Study 2017 (GBD 2017), 2018] study estimated that the 12-month prevalence for any MNS disorder in South Africa in 2016 was 15.9% (excluding epilepsy and intellectual disability) and 16.2%, including epilepsy and intellectual disability (Table 4; GBD 2017, 2018). Based on an uninsured South African population of over 46.4 million, we have estimated that there were approximately 7.5 million uninsured individuals living with a MNS disorder in 2016. With total MHIAs for the country reported as 88 444, and an average readmission rate of 24.2% across all hospitals in South Africa; we can crudely model that approximately 0.89% of the uninsured South African population requiring care received some form of public inpatient mental healthcare during the 2016/17 FY. Similarly, with total MHOVs reported as 567 277, we can crudely model that approximately 7.5% of the uninsured South African population requiring care received some form of public outpatient care during this period. These figures are crude in that they do not take into account the impact of multiple outpatient visits for the same MHCUs.

**Table 4:** Prevalence of MNS disorders, epilepsy and intellectual disability and proportions of target population(s) accessing inpatient and outpatient mental healthcare, South Africa

Cause	Prevalence (2016; Global Burden of Disease Collaborative Network, 2018)
Idiopathic developmental intellectual disability	1.7%
Epilepsy	0.6%
Schizophrenia	0.2%
Alcohol use disorders	1.6%
Drug use disorders	0.7%
Depressive disorders	3.9%
Bipolar disorder	0.6%
Anxiety disorders	3.8%
Eating disorders	0.2%
Autistic spectrum disorders	0.8%
Attention-deficit/hyperactivity disorder	1.2%
Conduct disorder	0.8%
Total: mental and substance use disorders	15.9%
Total: mental and substance use disorders, epilepsy and intellectual disability	16.2%
Total uninsured population (South Africa), 2016/17	46 392 634
Modelled estimate: total population (uninsured) living with mental and substance use disorders, epilepsy and intellectual disability (2016/17)	7 534 125
Total: inpatient mental health admissions, 2016/17	88 444
Modelled estimate: total inpatient mental health admissions that were readmissions, 2016/17	21 404
Modelled estimate: % of uninsured South Africans living with mental and substance use disorders, epilepsy and intellectual disability (2016/17) that have accessed inpatient care (2016/17)	0.89%
Total: outpatient mental health admissions, 2016/17	567 277
Modelled estimate: % of uninsured South Africans living with mental and substance use disorders, epilepsy and intellectual disability (2016/17) that have accessed outpatient care (2016/17)	7.5%

## Discussion

To the best of our knowledge, this is the first study to characterize the public health system expenditure on mental health services in South Africa and document the resources and constraints to the mental health system by service-level and province; achieving one of the highest sample sizes of any costing study conducted for mental health in LMICs (WHO, 2018b). This study builds on a situational analysis of the policy context, strategic needs, barriers and opportunities for sustainable financing for mental health in South Africa (Docrat *et al.*, 2019) by providing policy-makers with the necessary information to identify priorities and resources for mental health service scale-up to make progress towards the country’s progressive MHPF and achieve better mental health outcomes for South Africans. Furthermore, while the study was not able to report on all health system inputs due to data limitations, it was able to report on HR for mental health, access to essential medicines, infrastructure and resourcing. This article set out to propose and apply a methodology that addresses a number of key information gaps for LMICs contemplating mental health system reform. These gaps have thus far limited efforts to scale-up integrated mental healthcare and achieve global health and development targets. Understanding the variation in health system resources and constraints within countries represents the first step in a rational approach to planning for the implementation of mental health reforms. This study has attempted to address these constraints by providing data regarding national mental health resources, costs and treatment coverage in South Africa—both to provide a baseline for planned UHC investments and to illustrate methods for this task in other LMICs.

South Africa’s public mental health expenditure represented an estimated 5.0% of the total public health budget in the 2016/17 FY. Provincial expenditure on mental healthcare represented between 2.1% and 7.7% of provincial health budgets, with the majority of provinces (six of the nine) spending <5% of their health budgets on mental healthcare. It has been estimated that to match the most comprehensive mental health systems in the world, countries should expect to allocate up to 10% and a minimum of 5% of the total health budget to mental health (Chisholm *et al.*, 2006). Although South Africa is spending close to the lower target on the delivery of mental healthcare, modelled estimates revealed that approximately 0.89% and 7.5% of the uninsured South African population requiring care received some form of inpatient and outpatient care, respectively—suggesting the treatment gap for mental disorders, epilepsy and intellectual disability in South Africa is close to 92%.

A global scoping review of the availability of resources for mental health found that across LMICs, not only are resources limited for mental health service provision, but they are inequitably distributed and inefficiently used (Saxena *et al.*, 2007). The findings for South Africa confirm this, with huge disparities between provinces in the allocation of mental health resources. Per capita expenditure (uninsured) on inpatient and outpatient mental health services ranges from USD3.5 to USD22.1 between provinces. There are huge disparities in mental health personnel across provinces with the availability of psychiatrists ranging from 0.08 to 0.89 per 100 000 uninsured population. These disparities need to be rectified with a more consistent, evidence-based approach to planning. This study has confirmed that the majority of public sector psychiatrists is concentrated in the urban provinces which is consistent with existing evidence (Burns, 2010). Yet, mental health workforce targets for psychiatrists for the southern sub-Saharan region suggest that 1.9 psychiatrists per 100 000 will be needed by 2050 (Charlson *et al.*, 2014). Given the low absolute levels of psychiatrists currently working in the public sector in South Africa, it is unlikely that sufficient psychiatrists will be available to service mental health needs. We know that nurses represent the backbone of PHC services and in the absence of widespread access to psychiatrists, present a key resource to mental health service delivery. The analysis found that there is a high coverage of both professional and specialist nurses across the provinces, reporting a coverage of 80 per 100 000 and 27.3 per 100 000, respectively. Provinces must commit to ensuring that—where shortages have been identified—plans and resources are targeted to ensure generalists, nurses and community health workers are trained in task-shifted approaches for the delivery of mental healthcare, including care for children and adolescents, and private providers are contracted where no psychiatrists are envisaged to be available in the public health system.

In addition to the inequitable distribution of mental health resources across South Africa, the resources are not optimally used. The findings revealed that inpatient care forms the main source of care, comprising 86% of mental health expenditure, with specialized psychiatric hospitals comprising 45% of the total cost. Due to the limited number of mental health indicators to monitor service delivery at the PHC level, expenditure at this level of care may be underestimated but is unlikely to change the overall estimate of expenditure greatly. This is a reflection of the historical hospi-centric legacy of the country. Although Limpopo and Mpumalanga both spent larger proportions on outpatient care when compared with other provinces, this is due to the complete absence of any specialized psychiatric hospitals in Mpumalanga and a very limited number in Limpopo. Although global recommendations have urged countries to redistribute existing hospi-centric mental health budgets towards more efficient and effective uses in community-based settings—in the absence of adequate community-based services in South Africa, investments in psychiatric and hospital-based care must be maintained in the short-term, while concurrent bridge funding is earmarked to support capital investments to establish community-based services across the country.

Acknowledging that most mental disorders have their onset before the age of 18 years and approximately 38% of the population falls in this age bracket, this study has revealed an exceptional gap in terms of the service availability for children and adolescents in South Africa (Kessler *et al.*, 2005; STATS SA, 2017; WHO, 2018a). Only 6.8% of MHIA and 5.8% of MHOV were for patients below 18 years; and only three provinces reported the existence of public-sector child psychiatrists. The mental health of those aged between 10 and 19 years can profoundly impact their future health, social and economic circumstances as adults, particularly in contexts of poverty and vulnerability (Kessler *et al.*, 2005; WHO, 2018a). Improving and protecting adolescent mental health requires early detection, through routinized mental health screening, and early treatment both with and without pharmacological intervention (WHO, 2018a). Further, mental health prevention and promotion campaigns are critical at this age, to capacitate adolescents with resilience to cope with difficulties and avoid risk-taking behaviours (WHO, 2018a). Although efforts were made to cost DOH subsidized mental health promotion and prevention campaigns, none could be identified where funding had been directly provided by the DOH. Yet, most health districts who contributed to this study reported a considerable number of self-initiated campaigns, delivered without budgetary support in primary care settings, in response to the needs identified within their communities. There is a critical need for accelerated action for improved access to treatment and targeted mental health prevention and promotion for adolescents.

Across all hospital-levels, the duration of MHIAs was substantially longer than admissions for all conditions. At the district hospital-level, mandated as the first point of contact for MHCUs, clear contradictions to the recommendations of both the MHPF and the MHCA emerged (DOH, 2002, 2013; Petersen *et al.*, 2016). These hospitals are assigned the responsibility of ensuring that MHCUs are assessed and provided with ongoing referrals to more specialist treatment within a 72-h period, yet, this study revealed that mental health patients admitted to district hospitals spend >8 days as an inpatient at this level of care and the majority of facilities do not meet care requirements (DOH, 2002, 2013; Petersen *et al.*, 2016). At higher levels of care, even larger differences are seen between the admission lengths for all admissions, compared with mental health admissions, reflecting an absence of effective referral mechanisms for the complex long-term care needs of MHCUs.

Similarly, adequate attention must be paid to the potential savings that may yield from reducing readmission rates for all hospitals which cost the health system USD112.07 million. This is stark when compared with the total PHC-service for mental health costing USD45.3 million during the same period (excluding PHC services provided by NGOs). Readmission rates have been used as a proxy for relapse or complications following inpatient admission, and serve to indicate premature discharge, quality of care received prior to discharge or a lack of co-ordination and continuity of care with outpatient services post-discharge (Donisi *et al.*, 2016). Given the long length of inpatient admissions in South Africa, the high rates of readmission are likely a result in systemic failures when patients transition from hospitals to the next source of care within the community (Amoah and Mwanri, 2016).

This study confirmed that medications prescribed for the first-line treatment of several severely disabling MNS disorders, including depression and bi-polar disorder, were among the most frequently stocked out. Further, despite being listed in the STGs and EMLs, a number of mental health medications are NRA at level(s) of care for which the guidelines mandate their use, which points to a need to update the guidelines or improve their implementation (DOH, 2012a,b). The unavailability of medications at PHC-level may be partly due to the unavailability of doctors and healthcare workers with advanced psychiatric training authorized to initiate treatment, which speaks to the need to move towards nurse-initiated prescribing of psychotropic medication, particularly for depression and anxiety disorders.

There are a number of study limitations which should be noted. Firstly, most facilities that contributed to this study were unable to report a diagnostic disaggregation of inpatient and outpatient caseloads, and could not provide the average length of inpatient admissions for mental health patients, readmission rates and referral pathways post-discharge without extensive reviews of their patient records over a 1-year period. Secondly, tracking health personnel is instrumental in the delivery of mental health services in the country and critical in order to determine access to care and address shortages; yet the current staffing database of the DOH could not identify specific cadres of specialists or specialist nurses, making estimates of the availability of psychiatrists limited to those facilities and districts that completed primary data collection and estimates of the availability of specialist nurses with advanced psychiatric training indistinguishable from those with other advanced training in other areas. Thirdly, although this study described the availability of psychotropic medications, there was discordance between the information received from direct facility input, which reported a significant number of stock-outs, and stock-out reports generated by the NDOH. For this reason, little remains known about the underlying reasons for these stock-outs, and further interrogation is required. In addition, the NDOH must ensure that the centralized monitoring of psychotropic medications is improved to ensure it reflects the realities being faced by facilities on the ground. Despite attempts to cost expenditure on contracted hospitals and NGOs, not all provinces were able to provide expenditure on these data. Upcoming research will include the mapping out of residential and day care facilities, understanding population needs and existing resourcing for this level of service delivery; this has been identified as a priority for the South African government. The study did also attempt to collect data on the training of health personnel, a key strategy to strengthen primary care in terms of skills and competencies; however, a large number of facilities were not able to report accurately or comprehensively on training received by their personnel. Furthermore, data on referrals and the continuity of care for mental health users were not comprehensively available from reporting facilities to understand access to specialist services.

With a baseline understanding of current expenditure and coverage for mental health services in South Africa, future research should focus on determining the cost of scaling up mental healthcare in keeping with international cost-effective recommendations and potential system savings that may be incurred as a result. Furthermore, while this study provides a cross-sectional snapshot of health system utilization for MNS, longitudinal studies will help elicit an understanding of trends over time to monitor progress. Although global recommendations call for the integration of mental health within PHC, there remains a critical need to strengthen information systems for mental health to ensure that the goals of the MHPF are met and mental health services are embedded within the country’s plans for UHC through the NHI Scheme. Fiscal constraints and multiple competing health demands require a re-orientation away from hospi-centric models of care to allow for increased decentralization of services. Investments in primary- and community-based mental healthcare not only improves the efficiency of the health system and attempts to address the high rates of readmissions in hospitals but also allows for increased access to mental health services and the actualization of South Africa’s commitment towards deinstitutionalization.

## Conclusion

Despite South Africa’s supportive legislative and policy environment, in the absence of explicit tracking of resources and essential health system inputs, meeting the goals of the South African mental health policy and commitments for UHC more broadly, will remain a challenge. Whilst acknowledging limitations in health information systems to track dedicated health expenditure and the delivery of services, this study offers a nationally representative reflection of the state of mental health spending and elucidates inefficiencies in the system that may be addressed to increase the resource envelope for the delivery of critical mental health services within an integrated primary care model.

*Ethical approval.* This study made use of secondary data and collected routine health services data pertaining to mental health service delivery in South Africa from the NDOH and nine PDOH. No direct access to any facilities was required and no data that were collected in this study contained any patient identifiers. Ethics approval was obtained from the University of Cape Town Human Research Ethics Committee (HREC 744-2017) and from PHRC in each province. Written permission for this study was also provided by Provincial Heads of Health.

## Supplementary Material

czz085_Supplementary_Data
