# Supplementary material for: Mental health system costs, resources and constraints in South Africa: a national survey
Source: Health Policy Plan. 2019 Sep 23;34(9):706–19. doi: 10.1093/heapol/czz085 (PMC6880339; doi:10.1093/heapol/czz085)
Supplement: czz085_Supplementary_Data [file czz085_supplementary_data.zip › czz085-Suppl_data/Supplementary_Figure 2.docx]

**Supplementary Online Figure 2: MNS Disorder Medications most frequently stocked out by level of the health system**
