# Supplementary material for: Mental health system costs, resources and constraints in South Africa: a national survey
Source: Health Policy Plan. 2019 Sep 23;34(9):706–19. doi: 10.1093/heapol/czz085 (PMC6880339; doi:10.1093/heapol/czz085)
Supplement: czz085_Supplementary_Data [file czz085_supplementary_data.zip › czz085-Suppl_data/Supplementary_Table 1.docx]

**Supplementary Online Table 1: Secondary Data Sources, Indicators and Definitions**

| **Indicator Name** | **Source** | **Definition** | **Period** |
| --- | --- | --- | --- |
| Mental health clients total | DHIS | Total outpatient visits for mental health conditions | April 2016-March 2017 |
| Mental health clients 18 years and older | DHIS | Total outpatient visits for mental health conditions for clients 18 years and older | April 2016-March 2017 |
| Mental health clients under 18 years | DHIS | Total outpatient visits for mental health conditions for clients under 18 years | April 2016-March 2017 |
| Mental health admissions total | DHIS | Total number of clients admitted for mental health conditions (both voluntary and involuntary) | April 2016-March 2017 |
| Expenditure per patient day equivalent | HST | A composite indicator which measures the average cost per patient day equivalent (PDE) calculated by dividing the total expenditure of a hospital by the number of PDEs. PDE is calculated by adding the number of inpatients, plus half the number of day patients, plus one third the number of outpatients and emergency room visits as recorded in the DHIS | April 2015-March 2016 |
| PHC expenditure per headcount | HST | Provincial expenditure on: clinics, CHCs, community-based services and other community services, nutrition, HIV plus local government expenditure on PHC divided by PHC headcount from DHIS | April 2016-March 2017 |
| Average Length of Stay (all patients) | NDOH | Average length of inpatient admission calculated in days for all patients, regardless of diagnosis. | 2017 |
| Posts filled by health worker | NDOH | PERSAL human resource database indicating total posts filled for all cadres of health workers organized by facility | 2018 |
| DHIS = District Health Information System, HST = Health Systems Trust, NDOH = National Department of Health | | | |
