# Supplementary material for: Mental health system costs, resources and constraints in South Africa: a national survey
Source: Health Policy Plan. 2019 Sep 23;34(9):706–19. doi: 10.1093/heapol/czz085 (PMC6880339; doi:10.1093/heapol/czz085)
Supplement: czz085_Supplementary_Data [file czz085_supplementary_data.zip › czz085-Suppl_data/Supplementary_Table 2.docx]

**Supplementary Online Table 2: Proportion of Mental Health Inpatient and Outpatients by Health-facility type**

|  | Number of Facilities | Total Mental Health Outpatients | Total Mental Health Inpatients | % of Mental Health Outpatients | % of Mental Health Inpatients |
| --- | --- | --- | --- | --- | --- |
| Health Post | 16 | 404 |  | 0.02% |  |
| Mobile* | 801 | 39888 |  | 2.05% |  |
| Clinic* | 3406 | 905618 |  | 46.44% |  |
| Community Day Center* | 65 | 114293 |  | 5.86% |  |
| Community Health Centre* | 276 | 480837 |  | 24.66% |  |
| District Hospital | 251 | 167083 | 33074 | 8.57% | 44.33% |
| Mental Health Centre | 1 | 17669 | 296 | 0.91% | 0.40% |
| National Central Hospital | 9 | 1775 | 3786 | 0.09% | 5.07% |
| Provincial Tertiary Hospital | 18 | 30102 | 4742 | 1.54% | 6.36% |
| Regional Hospital | 47 | 114048 | 14629 | 5.85% | 19.61% |
| Specialised Psychiatric Hospital | 24 | 58261 | 17304 | 2.99% | 23.19% |
| Other Specialized Hospital | 6 | 19963 | 778 | 1.02% | 1.04% |
| *Definitions of Primary Health Care Facilities [32]  ***Clinic****: Render a nurse driven primary health care service at clinic level including visiting points, mobile- and local authority clinics. a range of primary health care services is provided here and that is normally open eight or more hours a day based on the need of the community to be served.*  ***Mobile****: Mobile clinics offer services such as pregnancy tests, ultrasounds, diabetes and blood pressure testing. Mobile units are often, but not always staffed with general practitioners and nurses. The mobile clinics aim to visit a community in a sub-district twice a month, but generally go to where the demand is the highest*  ***Community Day Centers****: Community day centers usually operate between 07:30 am to 16:00 from Monday to Friday. Services offered are for women’s health which include family planning, antenatal care and termination of pregnancy (TOP); an integrated chronic disease management consisting of non-communicable and communicable disease and mental health services which will include antiretroviral (ART) services; minor surgical procedures which includes the medical male circumcision (MMC) procedure; dietetics; chronic medicine collection (CDU); orthopaedic nursing outreach; wound care and dermatology and pharmacy services.*  ***Community Health Centre:*** *Rendering a primary health service with full-time medical officers in respect of mother and child, health promotion, geriatrics, occupational therapy, physiotherapy, psychiatry, speech therapy, communicable diseases, and mental health, amongst others. Services include 24-hour maternity, accident and emergency services and beds where health care users can be observed for a maximum of 48 hours and which normally has a procedure room but not an operating theatre.* | | | | | |
