# Supplementary material for: Mental health system costs, resources and constraints in South Africa: a national survey
Source: Health Policy Plan. 2019 Sep 23;34(9):706–19. doi: 10.1093/heapol/czz085 (PMC6880339; doi:10.1093/heapol/czz085)
Supplement: czz085_Supplementary_Data [file czz085_supplementary_data.zip › czz085-Suppl_data/Supplementary_Table 3.docx]

**Supplementary Online Table 3: District Hospital Infrastructure for Mental Health Inpatients**

| **Dimensions of District Hospital Infrastructure** | EC | FS | GT | KZN | LP | MP | NC | National |
| --- | --- | --- | --- | --- | --- | --- | --- | --- |
| Proportion of District Hospitals included in the sample (%) | 69% | 100% | 80% | 91% | 84% | 100% | 100% | 84% |
| Proportion of sampled District Hospitals designated for 72-hour Assessments by the Mental Health Care Act (2002) (%) | 62% | 100% | 88% | 81% | 96% | 87% | 82% | 84% |
| Proportion of sampled District Hospitals with Inpatient Psychiatric Unit (%) | 14% | 24% | 50% | 25% | 19% | 4% | 0% | 18% |
| Proportion of sampled District Hospitals reporting that Mental Health Inpatients are kept together with non-mental health patients in a general ward (%) | 51% | 0% | 63% | 81% | 78% | 96% | 82% | 62% |
| Proportion of sampled District Hospitals reporting that Adult Mental Health Inpatients are kept separate from Adolescent Mental Health Inpatients (%) | 8% | 0% | 25% | 3% | 0% | 30% | 64% | 13% |
| Proportion of sampled District Hospitals reporting that Male Mental Health Inpatients are kept separate from Female Mental Health Inpatients (%) | 70% | 100% | 75% | 63% | 96% | 87% | 36% | 78% |
| EC = Eastern Cape, FS = Free State, GT = Gauteng, KZN = Kwa-Zulu Natal, LP = Limpopo, MP = Mpumalanga, NC = Northern Cape  NB: The North West and Western Cape province(s) were unable to provide complete data regarding district hospital infrastructure for mental health inpatients, and have therefore been excluded. | | | | | | | | |
